# Surgical Resection of Tracheal Diverticulum with Haemoptysis as Unusual Presentation

**DOI:** 10.1155/2019/3828197

**Published:** 2019-02-27

**Authors:** Leonardo Toscano, Daniel Terra, Siul Salisbury, Nicolas Arechavaleta

**Affiliations:** ^1^Department of Thoracic Surgery, Army Forces Central Hospital, Uruguay; ^2^Department of Thoracic Surgery, Clinical Hospital, University of Republic, Uruguay; ^3^Department of Endoscopy Thoracic Surgery, Maciel Hospital, Uruguay

## Abstract

Tracheal diverticulum is defined as an air cyst located on the lateral wall, congenital or acquired. Most of them are asymptomatic, incidentally found on CT. The common symptoms are chronic cough, stridor, or recurrent respiratory infections. Asymptomatic diverticulum requires no treatment and managed conservatively while surgical excision is indicated in cases of local complications or symptom permanence. We report a case of tracheal diverticulum presented with haemoptysis, a rare symptom. The diagnosis was made with a CT that shows a 15 mm air image on the right lateral trachea wall. Due to symptoms' persistence, we decide to perform surgery with a good outcome.

## 1. Introduction

Tracheal diverticulum or tracheoceles are air cavities located adjacent to the trachea. It can be a single or multiple invaginations of the tracheal wall. They are usually incidentally diagnosed during a CT. The incidence of these cysts is as high as 4% in the general population, most of them being asymptomatic.

The differential diagnosis includes faringoceles, laryngoceles, Zenker diverticulum, pulmonary apical hernias, blebs, pneumomediastinum, or bullae [1–4].

We present a tracheal diverticulum with haemoptysis as the first symptom, an unusual initial presentation.

## 2. Case Report

A 26-year-old male patient, who is asthmatic and being treated with montelukast, presented with recurrent respiratory tract infections and repeated episodes of haemoptysis.

**Figure 1:**
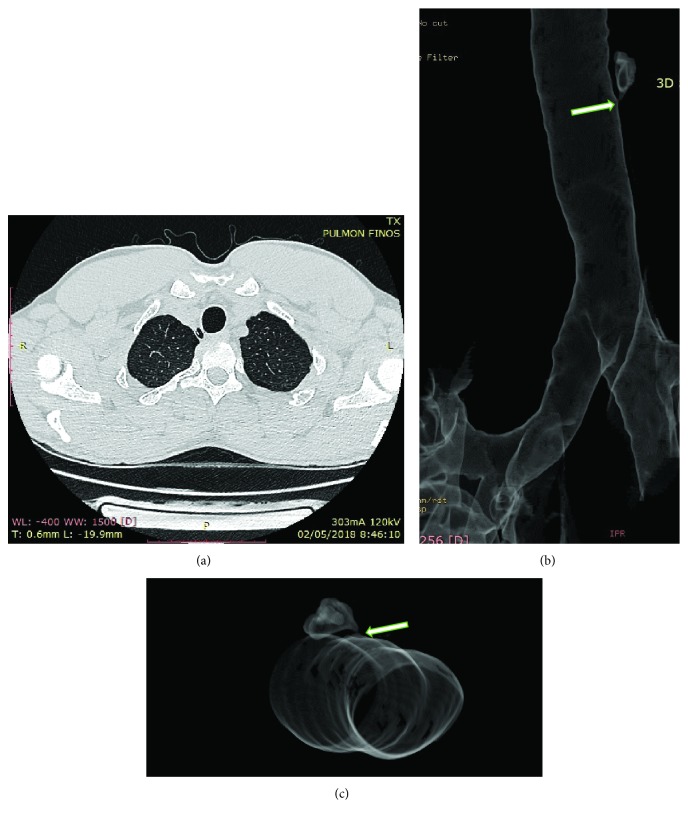
The axial CT (a) shows right paratracheal air cysts, measuring 15 mm in the right posterolateral side of the trachea at the level of C6. On 3D reconstruction (b, c), the white arrow shows communicating channel with the tracheal lumen.

There was no evident lesion more than a small depression in the mucosa, but no connecting duct was revealed in the bronchoscopy. The CT scan showed a right paratracheal well-defined air image immediately above the thoracic inlet (Figure 1). It was 15 mm in size and had a close relation with the posterior right trachea wall at the level of C6. There was no fluid inside the cavity and no sign of inflammation.

**Figure 2:**
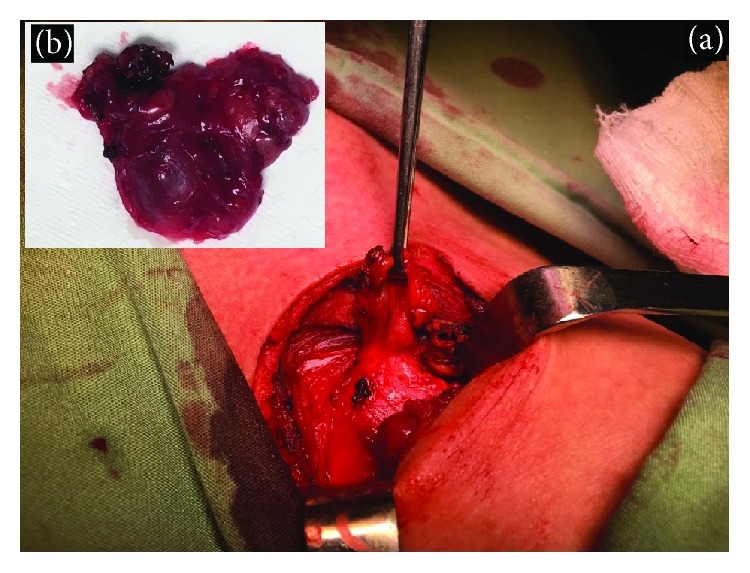
(a) The diverticulum is identified, exposed, and resected. The posterior membrane was sutured with absorbable thread. (b) The specimen was sent to the pathologist that confirmed that it was a benign cyst.

Due to the persistence of the symptoms, we decided to perform surgery to resect the tracheal diverticulum. We made a lower transversal anterior cervical incision, exposed the trachea, and carefully identified the cyst on the right side. The lesion was identified behind the trachea-oesophageal groove connected with the trachea (Figure 2). The laryngeal recurrent nerve was extremely adhered to the cyst but was freed. A complete resection was performed and then sutured with absorbable thread in the posterior membrane. The patient was discharged 72 hours after surgery with minor dysphonia. In the follow-up, two months after surgery, the patient was asymptomatic.

## 3. Discussion

Rokitansky, in 1838, was the first to describe tracheal diverticulum [2], with very few cases being reported since [5]. Even though the autopsy prevalence is near 1% (0.3% in children) [1, 4, 5], the CT incidence is about 4% in the healthy population [6].

Tracheal diverticulum is defined as one or multiple sacs that have originated from the trachea wall. Similar to pharyngeal diverticulum, which is more common, it may have the same pathogenic origin [7]. To assume this definition, it is necessary that there is communication between the sac and the trachea, which is not always present.

Another possible classification could define them as congenital or acquired. The first one is extremely rare and is a consequence of a malformation on the tracheal arcs [8].

The acquired form is secondary to an increase in airway pressure that makes a protrusion into a weak tracheal zone [8]. The most common cause of acquired diverticulum is cough, which is leading to the proposition by Goo et al. [4] that this is not just associated with COPD but instead is a sign of this disease.

The acquired cysts appear more commonly on the right side at the junction between the posterior membranous wall and the cartilages, more frequently at the thoracic inlet [5, 9]. They are larger than the congenital form and they have significant communication with the tracheal lumen. Despite these considerations, they can appear at any level along the trachea.

Polat et al. reviewed more than 8000 CT scans and found no difference between the aforementioned cysts and age; a mean age of 55 ± 16. There is no consensus about the distribution in terms of gender. The most common localization was the upper trachea (98%) and only 1.3% had more than one cyst. Most of them (97%) were on the right with a mean diameter of 5.5 mm ± 3.8 (from 1 to 23 mm). They demonstrated communication with the airway in only 16% [6] of the cases.

The right preferred location, like in our case, could be related to back support against the oesophagus on the left side, which constricts development on this side of the tracheal wall [4].

A tracheal cyst is often asymptomatic but a chronic cough, stridor, or recurrent infection may appear as symptoms. Less frequent symptoms like dysphagia, odynophagia, haemoptysis, hiccups, and/or burping could be present [7]. The main symptom in our patient was recurrent haemoptysis; this is a rare form of presentation.

The current available treatment options include observation, fine needle aspiration, and mediastinoscopy for aspiration as well as biopsy, thoracoscopy, thoracotomy, and mediastinoscopy for resection [10]. Endoscopy options are fulguration and endoscopic cauterization with a laser or electrocoagulation.

The resection is based on recurrence after aspiration, malignant degeneration, and intensity of symptoms [9, 11–13].

In our case, the recurrent infections and haemoptysis led us to advocate resection. Once surgery has been decided on, complete resection must be achieved. If it is not possible, partial resection and epithelium removal must be done.

Almost all the case series are about symptomatic patients, so there is not much evidence on asymptomatic or accidentally discovered cysts. The incidence of subsequent symptoms in initially asymptomatic patients is unknown, so there is no consensus about the treatment in this group of patients.
